# EHMTI-0046. The succession of aura symptoms: a prospective diary-based study

**DOI:** 10.1186/1129-2377-15-S1-D72

**Published:** 2014-09-18

**Authors:** M Viana, M Linde, G Sances, N Ghiotto, E Guaschino, M Allena, G Nappi, PJ Goadsby, C Tassorelli

**Affiliations:** 1 Headache Science Center, C. Mondino National Neurological Institute, Pavia, Italy; 2 Norwegian National Headache Centre Department of Neuroscience, Norwegian University of Science and Technology, Trondheim, Norway; 3 Headache Group – NIHR-Wellcome Trust Clinical Research Facility, King's College London, London, UK

## Introduction

Evaluation of clinical characteristic of non-hemiplegic migraine aura (NHMA), such as timing of different aura symptoms, is important as it might give us an insight in aura pathophysiology. Only one study assessed with a prospective diary the aura characteristics, reporting the different pattern of succession of visual and sensory symptoms in nine auras (Russell et al 1994).

## Aim

To evaluate the succession of individual symptoms of NHMA with a prospective diary-aided study.

## Methods

We recruited 136 consecutive patients affected by NHMA at the Headache Centers of Pavia and Trondheim. All the patients prospectively recorded the characteristics of three consecutive attacks in an ad hoc aura diary that included the time of onset and the end of each aura symptoms. We designated the first completing symptom as the first symptom (FS).

## Results

Of the 136 patients recruited so far, 44 completed the diaries during three consecutive auras for a cumulative number of 132 auras recorded. In 47 out of 132 auras there were at least two symptoms: in 16 auras (34%) the second symptom (SS) started simultaneously with the FS; in 14 auras (34%) the SS started during the FS; in 1 aura (2%) the SS started when the FS stopped, in 14 auras (30%) the SS started after a free interval of time after the end of FS (see Figure). In 11 auras there were 3 symptoms: in 1 aura (9%) the third symptom (TS) started simultaneously with the SS; in 6 auras (54%) the TS started during the SS; in 1 aura (9%) the TS started when the SS stopped: in 3 auras (28%) the TS started after a free interval of time after the end of SS.

**Figure 1:**
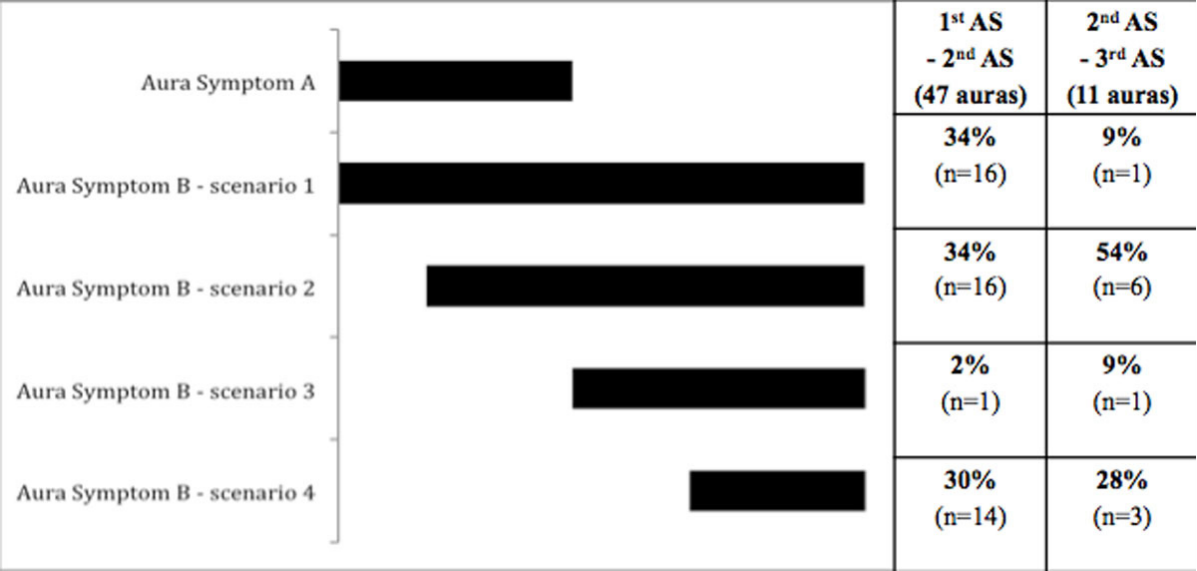


## Conclusions

It seems that in aura with multiple symptoms, the subsequent aura symptoms may either start simultaneously, during or after, the previous aura symptoms.

No conflict of interest.

